# Exclusive breastfeeding patterns in Tanzania: Do individual, household, or community factors matter?

**DOI:** 10.1186/s13006-020-00279-8

**Published:** 2020-04-22

**Authors:** Kwalu Samwel Dede, Hilde Bras

**Affiliations:** 1 Department of Political Ecology, International Institute of Social Studies, Erasmus University Rotterdam, Rotterdam, The Netherlands; 2 Department of Population Studies, Institute of Rural Development Planning, Dodoma, Tanzania; 3 Department of History, University of Groningen, Groningen, The Netherlands

**Keywords:** Exclusive breastfeeding, Infants, Child health, Nutritional status, Tanzania, Sub-Saharan Africa

## Abstract

**Background:**

Although there is a broad knowledge about exclusive breastfeeding among women in Tanzania, exclusive breastfeeding (EBF) remained lower than 50% for about 50 years since her independence in 1961. Previous research has mainly focused on either individual or household determinants of breastfeeding patterns. This study takes a holistic approach and examines the extent to which combined individual, household, and community factors matter in explaining exclusive breastfeeding patterns in Tanzania.

**Methods:**

A cross-sectional analysis was carried out using a nationally representative sample from the 2015/16 Tanzanian Demographic and Health Survey. The dependent variable was exclusive breastfeeding, defined as the proportion of infants below 6 months of age who were exclusively breastfed in the last 24 h. Univariable and multivariable logistic regression analyses were conducted to determine factors associated with exclusive breastfeeding.

**Results:**

In general, the rate of exclusive breastfeeding was 59%. Delivery in the short rainy season (95% Confidence Interval [CI] Adjusted Odds Ratio [AOR] 1.21, 2.65) was associated with higher odds of practicing exclusive breastfeeding. On the one hand, mothers aged between 15 and 19 years of age (95% CI AOR 0.36, 0.93), the average size of infants at birth (95% CI AOR 0.38, 0.80), whether postnatal check-up was attended by a doctor (95% C AOR 0.06, 0.46), and the infant’s age above 2 months (95% CI AOR 0.23, 0.53) were associated with lower odds of practicing exclusive breastfeeding. There was weak evidence (95% CI AOR 0.48, 1.05) that living in an urban area was associated with a reduced practice of exclusive breastfeeding.

**Conclusion:**

Breastfeeding rates are lower among young mothers, mothers whose husbands/partners decide on childcare, and mothers whose postnatal check-ups were conducted by doctors. Thus, breastfeeding programs and interventions need to focus more on young mothers, husbands/partners, and on training female nurses and midwives to increase the EBF rates. Women who tend to practice exclusive breastfeeding most often live in rural areas. There is an urgent need to understand why exclusive breastfeeding rates among urban women are lower.

## Background

WHO and UNICEF recommend exclusive breastfeeding (EBF) for the infant’s initial 6 months of life [1]. Studies on breastfeeding have shown that EBF rates in many countries are far from 100% and that EBF differs between and within populations. In the year 2016, for example, 23.3% of the mothers practiced EBF in Nigeria; 56.5% in Ethiopia, and 59% in Tanzania [2]. Tanzania is a relevant country to examine because still more than 40% of the infants are not exclusively breastfed and thus at risk of infectious diseases and malnutrition. We ask, what are the determinants of exclusive breastfeeding?

Many studies have examined the reasons for low rates of EBF by looking at individual factors such as the sex and age of the child, the age and educational level of the mother. Other studies have examined the impact of household factors such as household size and decision-making, including household socioeconomic status (SES) [3, 4]. Still, other research has hypothesized that the environment of breastfeeding mothers determines exclusive breastfeeding. Health facilities for maternal and childcare have also been found as essential factors [5]. The urban areas more often provide health facilities and services and conducible transport infrastructure compared to rural areas. That is why, in some cases, urban EBF rates are higher than rural rates [6, 7].

This study adds to the body of knowledge by examining EBF patterns in Tanzania using a holistic approach, including individual, household, and community factors. This work is the first to systematically explore how the combined individual, household, and community factors in Tanzania using DHS data. The results of the study are useful for professionals working in health-related fields such as Reproductive and Child Healthcare (RCH) units. The results are also relevant to policy and decision-makers who devise and implement feasible interventional plans.

### Explaining exclusive breastfeeding patterns: literature review and theoretical framework

In striving to understand patterns of exclusive breastfeeding, we take a holistic approach to examine the extent to which individual, household, and community-level variables are associated with EBF practices in Tanzania [8]. The individual-level factors are related to the educational level, marital status, and age of the mother; and the sex, birth order, and date of birth of the infant. The household-level factors include household size, spousal age, the partner’s educational level, and the number of other wives. Health facilities and services, and place of residence (rural or urban) constitute the community-level factors [8].

### Individual-level factors

The individual-level comprises mother and child characteristics. Mother characteristics that have been widely documented to influence exclusive breastfeeding are marital status, educational level, age, and occupation of the mother [9–16]. For example, Egata et al. [10] and Adugna [17] found in Ethiopia that mothers who did not practice EBF were often unmarried. Educational levels may also influence the likelihood of exclusive breastfeeding. A study in Northwest Ethiopia showed that mothers unable to read and/or write were three times more likely to offer breast milk to their infants for up to 6 months compared to mothers with secondary or higher educational levels [14]. According to them, educated mothers were more often employed and worked outside their homes and thus had less time for their infants [14]. Several studies report on the association of maternal age with breastfeeding practices [16–22]. For example, Naanyu’s research in Kenya found that breastfeeding duration was longer among mothers of 21–30 years and 31+ years old compared to younger mothers [20]. Younger-aged (< 20 years) mothers would have lacked the experience and right information on infant feeding.

Child characteristics such as sex, birth order, season of birth, and age may also affect breastfeeding practices. For example, Sefene et al. [14] showed that because of the custom of son preference in Ethiopia, male infants were two times more likely to be breastfed than female infants. In contrast, a study by Mosha et al. [23] in Morogoro, Tanzania, found that female infants were breastfed for a more extended period of time than male infants. The matrilineal kinship system of Morogoron society serves as an explanation for these differences. Similarly, studies in some Indian cultures found that female infants were given the mother’s breast milk for a more extended period than male infants. These gender differences widened with parity (higher birth order), higher parity boys received an even shorter period of breastfeeding [24]. Taylor et al. [24] in their study in the U.S., showed that experiences of breastfeeding of the previously born child determined the duration of breastfeeding of the current child. If the previous child was exclusively breastfed, then the chances that the next one was exclusively breastfed were higher. The age of the child can also influence the period of breastfeeding. According to the WHO, exclusively breastfed infants are less vulnerable to infectious diseases compared to infants introduced to complementary feeding before the age of 6 months [1]. Previous research has rarely examined how the season in which the child was born influences breastfeeding practices. We expect that children born in seasons with plenty of food can be exclusively breastfed because their mothers have higher chances of producing enough breast milk. Thus, we included this factor in our study.

### Household-level factors

Research has also indicated that household factors are associated with exclusive breastfeeding. Most studies have considered the effects of SES, or household wealth, and the size of the household. It is noted that mothers in low SES households experience poor nutritional status, which leads to insufficient production of breast milk for their nursed infants [25]. Others found that the nature and dwelling the mother lives in may influence exclusive breastfeeding rates [12]. According to them, poor households are associated with low economic status and likely also with poor nutritional status of the mother. Joshi et al. [25] note that mothers from wealthier quintiles had higher odds of EBF compared to mothers from the most deprived quintiles. They argue that the majority of the mothers from more affluent households have a better education level and thus high access to media and health services, which might have increased their awareness and, therefore, relatively conscious about exclusive breastfeeding. However, other studies indicate that mothers of higher SES do not practice EBF because they can afford to buy breast milk substitutes.

The size of the household also influences the rates of exclusive breastfeeding. Sefene et al. [14] found that Ethiopian mothers living in households with 5 + people were three times more likely to provide breast milk to their infants compared to mothers living in households with < 5 people. They argued that mothers in larger households are more likely to get assistance with household chores from other household members and can pay more attention to breastfeeding infants. Moreover, some studies have included decision-making on childcare, partner’s occupation, spousal age, and partner’s education [12, 25–27]. For example, mothers in Myanmar were found to be the sole decision-makers when it comes to breastfeeding their baby [27]. However, in some instances, elderly females advised breastfeeding mothers whether to continue or not [27].

### Community-level factors

Evidence shows that EBF patterns differ by the community and environmental factors such as place of residence and location of delivery. Concerning the setting, in several studies, mothers living in an urban area were found to have lower rates of EBF compared to rural mothers [24, 28, 29]. For example, the study of Asfaw et al. [30] in Ethiopia indicates that mothers from rural areas were about five times more likely to breastfeed their infants compared to urban mothers. Studies in Tanzania and Kenya show similar results [3, 4, 23, 31, 32]. For example, Victor et al. [31] found for Tanzania that slightly more than half of the rural mothers exclusively breastfed their infants compared to nearly 48% of the urban mothers. Likewise, a home delivery, as opposed to giving birth in the health facilities, lowers the duration of EBF because of a lack of appropriate professional advice on infant feeding practices. Findings show that giving birth in a health facility, and under the guidance of health professionals, increases the probability of practicing exclusive breastfeeding [11, 33–35].

### The setting: Tanzania

Tanzania is progressing in improving exclusive breastfeeding rates as determined by demographic and health indicators. For example, over a period of 13 years, EBF has increased from 23% in 1992 to 59% in 2015/16. This progress is a result of the establishment and implementation since 1974 of several nutritional and health interventions for mothers and newborns. Examples of such interventions are World Breastfeeding Week (WBW), Baby-Friendly Hospital Initiatives (BFHI), and national strategies on Infant and Young Child Feeding (IYCF) and Nutrition. Examples of the new approach include Health Support Program III (2008–2012), Primary Health Service Development Program (2007–2017), and the National Road Map Strategic Plan (2008–2018). They all focus on accelerating the reduction of maternal and newborn mortality through improved nutrition.

Administratively, Tanzania has 30 regions, 25 in the mainland area, and five in Zanzibar. These regions form nine different zones. Tanzania is linguistically a very diverse country with more than 126 languages spoken. However, not one language is spoken by a majority of the population. Swahili and English serve as the working languages in the country, with Swahili being the official national language. In most social groups, childcare and other household chores are considered the primary responsibility of women. Tanzania is among the African countries, which are most rapidly urbanizing. Tanzanian urbanization rates have increased by about 24% since its independence in 1961. In 2015, Tanzania had the sixth-highest population growth rate in the world; it is currently estimated at 3.3% [36].

## Methods

### Data collection

We use data from the eighth Tanzanian Demographic and Health Survey (TDHS) released in 2015/2016 to analyse factors influencing EBF patterns in Tanzania. The survey provides estimates of various health indicators, including fertility behaviour and preferences, marriage, sexual activities, family planning, and breastfeeding. The 2015/2016 DHS is a national representative survey, and it is useful for the present analysis because of the inclusion of variables on different levels of analysis. The data are collected by the MACRO (USAID) program, with the assistance of Tanzanian organizations. These organizations include the National Bureau of Statistics (NBS) and the Office of Chief Government Statistician (OCGS) of Zanzibar. The data set is accessible through the DHS website (https://dhsprogram.com/).

**Fig. 1:**
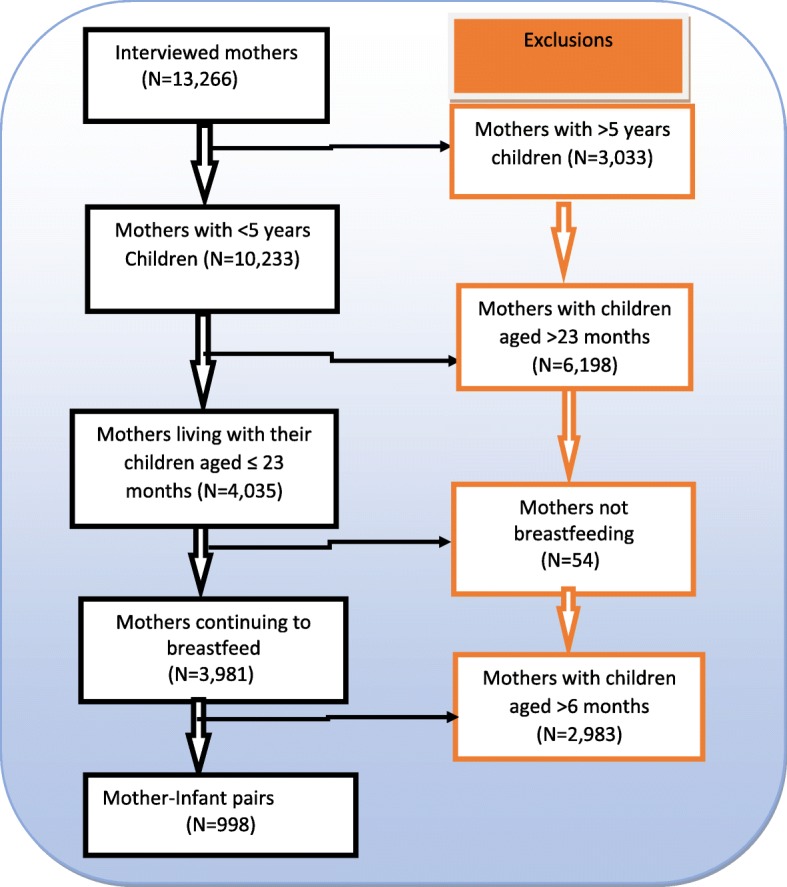
Steps used to construct the analytical sample, TDHS 2015/2016

The survey employed a national representative cross-sectional study design, using two-stage random sampling. In the first stage, 608 clusters from nine different zones as per delineated enumeration areas by 2012 Tanzania Housing and Population Census (THPC) were selected. About 13,000 households were selected, of which nearly 12,800 had an adult female resident. Of the occupied households, close to 12,600 were successfully interviewed, yielding a response rate of 98%. In the interviewed households, about 13,600 mothers of childbearing age (15–49 years) were identified for individual interviews. Nearly 13,300 women completed the interviews. The sample comprised only singleton mothers and excluded mothers with twins or triplets who do not often practice exclusive breastfeeding. In this study, we selected only women with children aged < 6 months to measure EBF, which left us with 998 women. Figure 1 summarizes the steps employed to construct the analytical sample.

### Measures

#### Dependent variable

The dependent variable in this study was EBF, which, by the WHO, is defined as the proportion of infants < 6 months of age who were exclusively breastfed in the last 24 h [37]. No other liquids, or even water, should be provided. However, drops and syrups consisting of vitamins, mineral supplements, or medicines, with a prescription by a medical specialist, can be given to the infant. The variable EBF is dichotomous, and its measurement follows the definition by the WHO (1 = Yes and 0 = No). It was constructed based on the mothers’ recall of what the infant was given as food during the last 24 h before the survey to reduce recall memory biases. Hence, all infants aged less than 6 months who were either given milk other than breastmilk and/or liquids, solid, and semi-solid foods were given a 0 on the EBF variable.

#### Independent variables

The independent variables include individual factors of the mother (age, years of education, marital status and occupation of the mother) and the child (sex, birth order, the season of birth, size of the child at birth, and age). Second, the household factors (household wealth index, partner’s education, decision-making regarding childcare, household size, partner’s occupation, and the number of other wives). We finally included the place of delivery and place of residence as predictor variables at the community level. The first column in Table 1 illustrates how each independent variable was measured. The season of birth was constructed based on the month in which the infant was born. Infants born in December through March were categorized as being born in the long rain season, those born in April through July as being born in the short rain season, and those born in August through November as being born in the dry season. These seasons apply to almost the whole of Tanzania.

**Table 1:** Univariable analysis of factors associated with exclusive breastfeeding, TDHS 2015/2016

Characteristic	N (%)	UOR	95% CI
Exclusive breastfeeding			
No	407(40.8)		
Yes	591(59.2)		
Age of the mother			
15–19 years	179(17.9)	0.69*	[0.49,0.97]
20–29 years	497(49.8)	1	Reference
30–39 years	271(27.2)	0.73	[0.73,1.34]
40–49 years	51(5.1)	0.75	[0.42,1.34]
Marital status			
Not married	173(17.3)	0.95	[0.68,1.33]
Married	826(82.7)	1	Reference
Occupation of the mother			
Casual/unskilled	378(37.9)	0.73*	[0.55,0.95]
Agriculture	536(53.7)	1	Reference
Small scale business	38(3.8)	0.52†	[0.27,1.01]
Skilled/professional	47(4.7)	0.99	[0.53,1.84]
Respondent worked past 12 months			
No	239(23.9)	0.83	[0.61,1.14]
In the past year	85(8.5)	1.94*	[1.17,3.20]
Currently working	480(48.0)	1	Reference
Have a job but on leave last seven days	195(19.5)	2.06***	[1.43,2.95]
Educational years of the mother			
No education	192(19.2)	0.85	[0.61,1.18]
1–7 years	647(64.8)	1	Reference
8–11 years	136(13.7)	1.26	[0.86,1.85]
12+ years	24(2.4)	0.51	[0.22,1.16]
Birth order of the child			
1st born	276(27.6)	0.88	[0.64,1.210]
2nd – 3rd born	366(36.7)	1	Reference
4th – 6th born	230(23.0)	1.38†	[0.98,1.94]
7th born and above	127(12.7)	0.81	[0.54,1.21]
Season of Birth			
Long rain season	138(13.8)	0.70†	[0.48,1.02]
Dry Season	575(57.6)	1	Reference
Short rain season	285(28.6)	3.72***	[2.66,5.19]
Sex of the child			
Male	519(51.9)	1	Reference
Female	480(48.1)	1.02	[0.79,1.31]
Size of the child at birth			
Very large	54(5.4)	0.66	[0.38,1.16]
Larger than average	161(16.1)	0.65*	[0.46,0.92]
Average	673(67.4)	1	Reference
Smaller than average	70(7.0)	0.75	[0.46,1.23]
Very small	34(3.4)	0.76	[0.38,1.54]
Don’t know	6(0.6)	1.49	[0.27,8.23]
The current age of the child			
< 2 months	560(56.1)	1	Reference
2–3 months	158(15.8)	0.34***	[0.23,0.49]
4–5 months	281(28.1)	0.11***	[0.08,0.15]
Household wealth index			
Poorest	265(26.6)	1	Reference
Poorer	205(20.6)	1.19	[0.82,1.73]
Middle	179(17.9)	0.99	[0.68,1.46]
Richer	204(20.5)	0.97	[0.67,1.40]
Richest	145(14.5)	0.92	[0.61,1.38]
Spouse years of education			
No education	123(12.3)	0.99	[0.67,1.48]
1–7 years	541(54.1)	1	Reference
8–11 years	154(15.4)	0.79	[0.55,1.14]
12+ years	8(0.8)	2.59	[0.46,14.58]
Missing	173(17.3)	0.92	[0.65,1.30]
Spouse age			
17–21 years	31(3.1)	0.92	[0.44,1.89]
22–41 years	642(64.3)	1	Reference
42–61 years	140(14.0)	1.10	[0.76,1.61]
62+ years	13(1.3)	0.57	[0.19,1.73]
Missing	173(17.3)	0.96	[0.86,1.35]
Decision making on childcare			
Mother	244(24.4)	0.94	[0.69,1.28]
Husband/partner	140(14.0)	0.63*	[0.43,0.91]
Joint decision	561(56.1)	1	Reference
Someone else	54(5.4)	0.96	[0.54,1.70]
Household size			
1–6 people	489(49.0)	1	Reference
7–16 people	459(45.9)	1.21	[0.93,1.57]
17+ people	51(5.1)	1.23	[0.68,2.22]
Number of other wives			
No co-wife	686(68.7)	1	Reference
One co-wife	108(10.8)	0.87	[0.58,1.31]
2+ co-wives	32(3.2)	0.81	[0.40,1.65]
Missing	173(17.3)	0.93	[0.66,1.30]
Spouse occupation			
Casual/unskilled	110(11.1)	0.80	[0.53,1.21]
Agriculture	498(49.8)	1	Reference
Small scale business	59(6.0)	1.10	[0.63,1.91]
Skilled/professional	158(15.9)	0.85	[0.59,1.22]
Missing	173(17.3)	0.90	[0.63,1.28]
Place of delivery			
Home	339(34.0)	1.04	[0.77,1.36]
TBA premises	4(0.4)	2.12	[0.23,19.25]
Public health facility	539(54.0)	1	Reference
Private health facility	106(10.6)	1.37	[0.89,2.11]
Other	10(1.0)	1.37	[0.46,5.19]
Person performed a p/natal check-up			
Doctor	26(2.6)	0.29**	[0.12,0.72]
Nurse/Midwife	254(25.5)	1	Reference
TBA/CHW	9(0.9)	1.34	[0.35,5.15]
Relative/Friend	21(2.1)	0.41†	[0.16,1.04]
Missing p/natal check-up	688(68.9)	1.29†	[0.96,1.73]
Distance from a health facility			
Big problem	450(45.10	0.95	[0.74,1.22]
Not a big problem	548(54.9)	1	Reference
Source of drinking water			
Unimproved	470(47.1)	1.25†	[0.97,1.62]
Improved	529(52.9)	1	Reference
Type of place of residence			
Urban	278(27.9)	0.64**	[0.49,0.85]
Rural	720(72.1)	1	Reference

Source: Generated from TDHS 2015/2016

* = *P* < 0.05; ** = *P* < 0.01 *** = *P* < 0.001; †=*P* < 0.10; *TBA* Traditional Birth Attendant, *CHW* Community Health Worker

### Analytical strategy

In this study, we considered the mother and infant characteristics at the individual level, household characteristics at the household level, and health facility services and place of residence at the community level. Frequencies and weighted percentages were presented along with univariable logistic regressions to examine how each of the independent variables relates to the outcome variable, exclusive breastfeeding. Both univariable logistic regressions and multivariable logistic regressions were conducted to assess the influence of the selected predictor variables on exclusive breastfeeding. Only variables that indicated evidence of significant association (*p* < 0.05) with exclusive breastfeeding were included in the adjusted model (see Table 2). All models were conducted at a confidence level of 95% using IBM SPSS statistics version 25. We reported the association between predictor variables and the outcome variable using both unadjusted odds ratios (UORs) and adjusted odds ratios (AORs).

**Table 2:** Multivariable analysis of factors associated with exclusive breastfeeding

Characteristic	AOR	95% CI
Age of the mother		
15–19 years	0.58*	[0.36,0.93]
20–29 years	1	Reference
30–39 years	1.24	[0.79,1.95]
40–49 years	0.79	[0.36,1.78]
Occupation of the mother		
Casual/unskilled	0.80	[0.49,1.31]
Agriculture	1	Reference
Small scale business	0.68	[0.30,1.56]
Skilled/professional	1.12	[0.52,2.40]
Respondent worked past 12 months		
No	0.91	[0.53,1.56]
In the past year	1.09	[0.61,1.94]
Currently working	1	Reference
Have a job but on leave last seven days	1.29	[0.82,2.02]
Birth order of the child		
1st born	1.16	[0.75,1.79]
2nd – 3rd born	1	Reference
4th – 6th born	1.04	[0.65,1.66]
7th born and above	0.57†	[0.31,1.07]
Season of Birth		
Long rain season	1.39	[0.87,2.22]
Dry Season	1	Reference
Short rain season	1.79**	[1.21,2.65]
Size of the child at birth		
Very large	0.68	[0.35,1.31]
Larger than average	0.54**	[0.36,0.80]
Average	1	Reference
Smaller than average	0.75	[0.41,1.36]
Very small	0.62	[0.26,1.44]
Don’t know	0.81	[0.14,4.83]
The current age of the child		
< 2 months	1	Reference
2–3 months	0.35***	[0.23,0.53]
4–5 months	0.11***	[0.08,0.17]
Decision making on childcare		
Mother	1.14	[0.79,1.65]
Husband/partner	0.62*	[0.39,0.97]
Joint decision	1	Reference
Someone else	0.96	[0.47,1.96]
Person performed a postnatal check-up		
Doctor	0.16**	[0.06,0.46]
Nurse/Midwife	1	Reference
TBA/CHW	1.34	[0.28,6.36]
Relative/Friend	0.35†	[0.12,1.01]
Missing p/natal check up	1.23	[0.87,1.74]
Source of drinking water		
Unimproved	1.088	[0.79,1.50]
Improved	1	Reference
Type of place of residence		
Urban	0.71†	[0.48,1.05]
Rural	1	Reference

Source: Generated from TDHS 2015/2016

* = *P* < 0.05; ** = *P* < 0.01 *** = *P* < 0.001; †=*P* < 0.10; *TBA* Traditional Birth Attendant, *CHW* Community Health Worker, *CI* Confidence Interval

## Results

Of the 998 mothers, about 50% were aged between 20 and 29 years, while nearly 54% were engaged in agricultural activities. Close to 37% of the mothers had a parity of 2–3 children, and about 58% gave birth in the dry season. In terms of the infants’ birthweight, 67% gave birth to normal weight babies. About 56% of the interviewed mothers had babies aged below 2 months.

Nearly a quarter (24%) of the mothers decided on the infant’s care, while about 26% received postnatal care from nurses or midwives. Approximately three quarters (72%) of the sampled mothers resided in rural areas. Generally, slightly more than half (59%) of all mothers practiced exclusive breastfeeding (see Table 1).

As illustrated in Table 1, the univariable logistic regression analysis indicated that mothers aged 15–19 years were about 30% less likely to practice EBF (UOR 0.69, 95% CI 0.49, 0.97) compared to mothers aged 20–29 years. Mothers who performed casual or unskilled work were about 0.7 (UOR 0.73, 95% CI 0.55, 0.95) times less likely to practice EBF compared to mothers engaged in agriculture activities. Mothers on leave during the last 7 days before the survey had about 2.0 (UOR 2.06, 95% CI 1.17, 3.20) times higher odds of EBF compared to mothers found working during the study. Findings also showed that mothers who delivered in the short rain season were about four times (UOR 3.72, 95% CI 2.66, 5.19) more likely to practice EBF than mothers who gave birth during the dry season.

Mothers whose babies had larger sizes at birth were 30% (UOR 0.65, 95% CI 0.46, 0.92) less likely to practice EBF compared to mothers whose babies had average sizes at birth. Likewise, mothers with infants aged 2–3 months were about 70% (UOR 0.34, 95% CI 0.23, 0.49) less likely to be exclusively breastfed compared to mothers with infants aged below 2 months.

Decision-making on childcare was an essential household level predictor of EBF. Mothers whose partners or husbands made the decisions about childcare were about 40% (UOR 0.63, 95% CI 0.43, 0.91) less likely to practice EBF compared to mothers who themselves decided on childcare. Postnatal care and place of residence also featured as significant predictors of EBF at the community level. Mothers attended by doctors during postnatal check-ups were about 70% (UOR 0.29, 95% CI 0.12, 0.72) less likely to practice EBF compared to mothers attended by nurses and midwives. Furthermore, urban mothers were about 40% (UOR 0.64, 95% CI 0.49, 0.85) less likely to practice EBF compared to rural mothers.

The multivariable logistic regression analysis showed that variables such as the age of the mother, the season of birth, and the size of the child at birth were significantly associated with EBF at a 95% confidence level. Moreover, the current age of the child, decision making on child care, and postnatal check-up were also significantly related to EBF (see Table 2). Contrary to univariable logistic regression results, the birth order of the child and the place of residence had a weak association with EBF at a 95% confidence level (Table 2). Mothers aged 15–19 years were associated with an almost 40% (AOR 0.58, 95% CI 0.36, 0.93) reduction in adjusted odds of practicing EBF compared to mothers aged 20–29 years. Infants born in the short rain season were about two times (AOR 1.79, 95% CI 1.21, 2.65) as likely to be exclusively breastfed compared to infants born in the dry season.

Also, infants with weight sizes above average at birth were about 50% (AOR 0.54, 95% CI 0.36, 0.80) less likely to be exclusively breastfed compared to infants with an average size at birth.

Exclusive breastfeeding decreased with a higher infant’s age. The results show that infants aged between 2 and 3 months were associated with about 60% (AOR 0.35, 95% CI 0.23, 0.53) reduction in the adjusted odds ratio of EBF compared to infants aged < 2 months.

Mothers whose husband/partner decided on childcare were about 30% (AOR 0.62, 95% CI 0.39, 1.05) less likely to practice exclusive breastfeeding.

Mothers attended by doctors during postnatal check-ups were about 80% (AOR 0.16, 95% CI 0.06, 0.46) less likely to practice EBF compared to mothers attended by nurses and midwives.

Concerning the place of residence, there was weak evidence that urban mothers were 30% (AOR 0.71, 95% CI 0.48, 1.05) less likely to practice EBF compared to rural mothers.

## Discussion

In this study, we attempted to establish whether some of the individual, household, and community factors, which we believed might influence EBF, had the expected impact on exclusive breastfeeding in Tanzania. Currently, (2015/16), Tanzania’s average EBF rate is 59% compared to a rate of below 30% in the 1990s [38].

Concerning the age of the mother, our findings indicate that the practice of EBF increased with the age of the mother, but again decreased for mothers aged 40 + years. Besides their experience, older women might be more occupied with household chores, including taking care of other children. Moreover, most of the urban and some of the rural mothers who gave birth at the age of 40 + years might be employees in either formal public or private sectors, where, in Tanzania, maternity leave extends for a maximum of 84 days. Similar studies show that young mothers have less breastfeeding experience as compared to older mothers [16, 19–22]. Infants who were born in the short rain season were two times more likely to be exclusively breastfed compared to those born in the dry season. The short rain season (April–July) is a period in which many parts of Tanzania experience plenty of food compared to the dry season (August–November). Thus, breastfeeding mothers may have had a higher probability of eating balanced diets, which enabled them a better production of breastmilk. Other studies have also observed that infants experience thirst during hot weather (dry season) and are more often given water to wet their mouths or quench their thirst [39].

It also became evident that the size of the child at birth and the current age of the baby were significantly associated with decreased rates of exclusive breastfeeding. A healthy baby is born with a weight of 2.5–4.0 kg and our study indicated that mothers whose children were born with sizes larger than average had odds ratios associated with a reduction in exclusive breastfeeding. This could be a result of maternal depletion during delivery and the mother’s possible inability to breastfeed during the recommended period (within an hour after birth). It is also possible that the largeness of the baby at birth, being closely related to a ceasarean section mode of delivery, may, in this way, reduce EBF rates. This study and other research indicate that the chances of the infant to be exclusively breastfed decreases with age [13, 37]. Reasons associated with this decline may include breastfeeding norms related to the size and the age of the child, and the fact that mothers returned to work after maternity leave.

Decision-making on child care, postnatal check-up, and place of residence were also significantly associated with the outcome variable. The findings indicate that mothers had odds ratios associated with decreased EBF if the husband/partner decided on childcare. Husbands who make the decisions on childcare may signal more patriarchal, male-dominated households, and thus may have a negative influence on exclusive breastfeeding [40]. Usually, the women are the ones who make decisions on childcare as this is a women’s domain in many African countries, including Tanzania. Similarly, if the doctors conducted mothers’ postnatal check-up compared to nurses, odds ratios for EBF were decreased. In the male-dominated, patriarchal culture of Tanzania, nurses and midwives perhaps advise mothers better on breastfeeding than doctors. Both doctors and nurses have received formal training. Doctors receive training in medicine (i.e., treatment of diseases through medical procedures and sometimes surgery) and at the same time, nurses receive training in caring for sick people besides knowing how to detect, treat, and manage various common cases. Thus, nurses may offer better advice on breastfeeding and encourage EBF more, because it has been more their field of attention, while doctors might have more distance to matters related to breastfeeding and thus may encourage EBF less.

Like Victor et al. [31], our findings indicate that rural mothers had higher chances of practicing exclusive breastfeeding. This can be explained by the fact that many urban mothers are employed in the public and private sectors. Therefore, they have less time for their babies. They more often work outside the home compared to rural mothers [3, 4, 23, 28–30, 32].

This study has several limitations. It became clear that not every factor included in this study was significantly associated with exclusive breastfeeding. For example, only one household variables (decision-making) had a significant association with the outcome variable in the multivariable analyses at a 95% confidence level. There is a possibility that there are other household-level explanatory variables that were significantly related to EBF, but which were not included in this study.

Conversely, we did not include other explanatory variables that may have had an association with exclusive breastfeeding. Such variables may consist of average community factors such as mothers’ educational level, marital status, and age. We also have to take into account that the variable season or month of birth is an indirect way to assess food intake. Finally, the data set used is a cross-sectional survey, so no statements about causality can be made.

## Conclusion

We identified six factors associated with EBF among women in Tanzania and found that EBF rates among young mothers are lower compared to older mothers. Breastfeeding programs and interventions need to focus more on young mothers to increase EBF rates. There is an urgent need for raising awareness among husbands/partners to breastfeeding mothers on the importance of exclusive breastfeeding. It is equally important to train female reproductive and child health (RCH) professionals who could counsel breastfeeding mothers. Nurses and midwives, in Tanzania’s patriarchal context, show that they do a much better job than doctors.

Furthermore, research is required to understand why the EBF rates among urban women are lower and what would be the consequences associated with this. The study is highly necessary because many formerly rural areas are now acquiring urban characteristics. Urbanization is not only a Tanzanian phenomenon but also occurs in other African countries with similar population characteristics.

Finally, future research should also consider the month or season of birth of the baby as this may say a lot about food availability and the nutritional status of the mother.

## Data Availability

Data used for this study are available at the DHS website: https://dhsprogram.com/.

## Abbreviations

AOR: Adjusted Odd Ratio
BFHI: Baby-Friendly Hospital Initiatives
EBF: Exclusive Breastfeeding
IYCF: Infant and Young Child Feeding
NBS: National Bureau of Statistics
OCGS: Office of Chief Government Statistician
OR: Odds Ratio
RHC: Reproductive and Child Healthcare
SES: Socioeconomic Status
TBA: Traditional Birth Attendant
TDHS: Tanzania Demographic and Health Survey
TPHC: Tanzania Housing and Population Census
UNICEF: United Nations International Children’s Emergency Fund
UOR: Unadjusted Odds Ratio
USAID: United States Agency for International Development
WABA: World Alliance for Breastfeeding Action
WBW: World Breastfeeding Week
WHO: World Health Organization
